# Population structure and genome-wide association analysis for frost tolerance in oat using continuous SNP array signal intensity ratios

**DOI:** 10.1007/s00122-016-2734-y

**Published:** 2016-06-18

**Authors:** Giorgio Tumino, Roeland E. Voorrips, Fulvia Rizza, Franz W. Badeck, Caterina Morcia, Roberta Ghizzoni, Christoph U. Germeier, Maria-João Paulo, Valeria Terzi, Marinus J. M. Smulders

**Affiliations:** 1 Council for Agricultural Research and Economics, Genomics Research Centre, Via San Protaso 302, 29017 Fiorenzuola d’Arda, PC Italy; 2 Wageningen UR Plant Breeding, Droevendaalsesteeg 1, NL-6708 PB Wageningen, The Netherlands; 3 Biometris, Wageningen UR, Droevendaalsesteeg 1, NL-6708 PB Wageningen, The Netherlands; 4 Julius Kühn Institut, Federal Research Centre for Cultivated Plants, Institute for Breeding Research on Agricultural Crops, 06484 Quedlinburg, Germany

## Abstract

***Key message*:**

***Infinium SNP data analysed as continuous intensity ratios enabled associating genotypic and phenotypic data from heterogeneous oat samples, showing that association mapping for frost tolerance is a feasible option.***

**Abstract:**

Oat is sensitive to freezing temperatures, which restricts the cultivation of fall-sown or winter oats to regions with milder winters. Fall-sown oats have a longer growth cycle, mature earlier, and have a higher productivity than spring-sown oats, therefore improving frost tolerance is an important goal in oat breeding. Our aim was to test the effectiveness of a Genome-Wide Association Study (GWAS) for mapping QTLs related to frost tolerance, using an approach that tolerates continuously distributed signals from SNPs in bulked samples from heterogeneous accessions. A collection of 138 European oat accessions, including landraces, old and modern varieties from 27 countries was genotyped using the Infinium 6K SNP array. The SNP data were analyzed as continuous intensity ratios, rather than converting them into discrete values by genotype calling. PCA and Ward’s clustering of genetic similarities revealed the presence of two main groups of accessions, which roughly corresponded to Continental Europe and Mediterranean/Atlantic Europe, although a total of eight subgroups can be distinguished. The accessions were phenotyped for frost tolerance under controlled conditions by measuring fluorescence quantum yield of photosystem II after a freezing stress. GWAS were performed by a linear mixed model approach, comparing different corrections for population structure. All models detected three robust QTLs, two of which co-mapped with QTLs identified earlier in bi-parental mapping populations. The approach used in the present work shows that SNP array data of heterogeneous hexaploid oat samples can be successfully used to determine genetic similarities and to map associations to quantitative phenotypic traits.

**Electronic supplementary material:**

The online version of this article (doi:10.1007/s00122-016-2734-y) contains supplementary material, which is available to authorized users.

## Introduction

Hexaploid oat (*Avena sativa* L.) is an important crop grown on nearly 10 million hectares worldwide (FAOSTAT 2015) mostly in temperate regions in a wide range of environmental conditions. Gene banks preserve about 80,000 accessions of cultivated oats and more than 20,000 wild oats. This germplasm can be considered a reservoir of potentially useful genes, to be exploited in pre-breeding and breeding programs (Lipman et al. 2005). An important trait for oat, to which much (pre-) breeding is devoted, is tolerance to frost. Among winter cereals, oat is in fact the most frost sensitive and its insufficient level of winter hardiness is one of the most important factors limiting the cultivation of winter oat in cold areas. In most of Europe therefore spring-sown oats are grown, but where winters are mild—like in the UK and in Southern Europe—winter oats, sown during the autumn, are preferred. Winter oats have a long growth cycle and early maturity, and give higher yields.

Winter hardiness can be defined as the ability to survive throughout the winter. Because of the wide range of stressful conditions that a plant may experience during the cold season, winter hardiness is a complex trait. Freezing temperature is the most relevant stress factor, although other stress situations, such as anoxia due to excess of water or to ice encasement, photoinhibition and mold infection may also occur (Stanca et al. 2003). The ability of overwintering plants to withstand cold is mainly based on an adaptive response, known as cold acclimation or hardening, activated during growth at low non-freezing temperatures. Frost tolerance—a complex trait influenced by Genotype by Environment (GxE) interaction—can be assessed through field evaluation methods, but these depend on the occurrence of the proper natural conditions to satisfactorily differentiate genotypes. Alternatively, artificial freezing tests have been used that measure the percentage of post-stress survival, LT50 (temperature at which 50 % of the population is killed), integrity of cell membranes after freezing (Rizza et al. 1994) or the chlorophyll *F*_v_/*F*_m_ ratio, that is the maximum quantum yield of the PSII photochemistry after freezing (Rizza et al. 2001).

Vernalization can be defined as the induction of the competence to flower by a prolonged exposure to winter cold. *Vrn*-*1* is the main locus responsible for vernalization sensitivity in cereals. In the currently accepted model for wheat and barley *Vrn*-*2* acts as a repressor of *Ft*-*1* (*Vrn*-*3*), which is an orthologue to the *ArabidopsisFlowering Locus T* responsible for flower initiation (Yan et al. 2006). During vernalization *Vrn*-*1* is progressively induced repressing *Vrn*-*2* and allowing induction of *Ft*-*1* (Yan et al. 2004; Trevaskis et al. 2006; Hemming et al. 2008; Chen and Dubcovsky 2012). Depending on vernalization requirement, sensitivity to photoperiod and frost tolerance the oat germplasm grown in temperate climates is usually divided into spring, winter and facultative types. Spring types tend to flower quickly, do not require vernalization and are often insensitive to photoperiod. Winter types show a strong vernalization requirement and are sensitive to short days. Facultative types do not require vernalization but show levels of frost tolerance similar to winter types. In barley the difference between spring, winter and facultative types has been mostly explained by molecular variation in *Vrn*-*H1* and *Vrn*-*H2*. A dominant allele of *Vrn*-*H1* determines the spring habit, while a homozygous recessive combination correlates with winter and facultative types (von Zitzewitz et al. 2005; Szucs et al. 2007). Allelic variation at *Vrn*-*H2* has been found to be responsible for the differences in vernalization requirement observed between winter and facultative types (Szucs et al. 2007). However, the distinction among the oat types does not appear so clear as in barley, since oat winter types sown in late spring are often able to flower and produce viable seeds (as observed in this study). In tetraploid wheat, *Vrn*-*1* null mutants were shown to flower very late but to produce normal flowers and seeds, suggesting that *Vrn*-*1* is not essential for wheat flowering and alternative flowering genes may exist (Chen and Dubcovsky 2012). In addition, an alternative pathway of flower initiation via the barley gene *HvFT3* was proposed by Casao et al. (2011).

*Frost Resistance*-*1* (*Fr*-*1*/*Vrn*-*1*) and *Frost Resistance*-*2* (*Fr*-*2*) are the main QTLs for frost tolerance in *Triticeae* and were mapped on the homoeologous group 5 chromosomes (Francia et al. 2004; Galiba et al. 1995). Both in barley and in wheat *Fr*-*1* co-segregates with *Vrn*-*1*; the frost tolerance attributed to *Fr*-*1* is therefore most likely a pleiotropic effect of *Vrn*-*1* (Limin and Fowler 2006; Dhillon et al. 2010). The locus *Fr*-*2* includes a cluster of more than 10 C-Repeat Binding Factor (CBF) transcription factors (Francia et al. 2007), which are known as regulators of COR (Cold-regulated) gene expression, playing a role in protection from frost damage (Park et al. 2015). In barley, an increase in copy number of *HvCBF2* and *HvCBF4* was suggested to be the causal polymorphism determining frost tolerance (Knox et al. 2010), although allelic variation at *HvCBF14* was found statistically significant as well (Fricano et al. 2009). Recently, Zhu et al. (2014) showed that bread wheat frost tolerance is significantly affected by the interaction between the locus *Vrn*-*A1* and *Fr*-*A2*. Two main allelic variants (S and T) were identified for *Fr*-*A2*, based on sequence polymorphisms in the region *CBF*-*A12*/*CBF*-*A15* and copy number variation of *CBF*-*A14*. An increased copy number of *Vrn*-*A1* enhanced frost tolerance but only when the T allele was present at the *Fr*-*A2* locus (Zhu et al. 2014). Mutations in the homoeologous genes *Vrn*-*B1* and *Vrn*-*D1* had a weaker effect (Zhu et al. 2014). In barley, genome-wide association study (GWAS) was successfully used for identifying main QTLs affecting frost tolerance (von Zitzewitz et al. 2011; Visioni et al. 2013; Tondelli et al. 2014). Another QTL affecting frost tolerance (*Fr*-*H3*) was identified on the barley chromosome 1H accounting for 48 % of the phenotypic variation in two double haploid populations originating from winter and facultative types (Fisk et al. 2013).

Knowledge on molecular pathways underlying frost tolerance, sensitivity to photoperiod and vernalization in oat is still poor. To this date, the main studies on oat winter hardiness were based on linkage mapping in bi-parental populations, using a limited number of AFLP, SSR or DArT markers. Recently, the Infinium 6K oat SNP array was developed (Tinker et al. 2014; Oliver et al. 2013). Moreover, a dense hexaploid oat consensus map based on 12 bi-parental populations, mostly originating from North American spring varieties, is now available (Chaffin et al. 2016). The availability of these tools together with new genetic populations—such as the wide European germplasm collection established by the AVEQ project (http://aveq.jki.bund.de/aveq)—opens new perspectives to the study of adaptive traits in oats.

In the present work we genotyped a diverse collection of European oats, including modern, old varieties and landraces, with potentially different degrees of sample heterogeneity. The approach we used is based on the analysis of SNP array signals as continuous intensity ratios, skipping the genotype calling step. This approach tolerates sample heterogeneity and avoids the effort and the potential (over-) interpretation errors involved in genotype calling, which is particularly challenging in allopolyploid genomes. The objectives of the work were: (1) to study genetic diversity in European oat germplasm, (2) to show the feasibility of GWAS based on continuous SNP signal ratios in a collection including heterogeneous samples for simple traits such as lemma color and hullessness, whose phenotypic variation is explained by few major genes, (3) to use GWAS and chlorophyll fluorescence data for identifying QTLs affecting frost tolerance in oat.

## Materials and methods

### Plant materials and DNA extraction

A collection of 138 oat accessions, originating from 27 European countries, was analyzed in the present study. It contains a few diploid *A. strigosa* accessions and 133 hexaploid *A. sativa* accessions, including landraces and cultivars used for spring and winter sowing and representing about 100 years of European oat breeding history. Further information about genebank holder, taxonomy, country of origin, growth habit and year of registration can be found in Online Resource 1.

To take into account intra-varietal genetic variability, young leaves from 10 plants for each accession were pooled and genomic DNA was extracted using a CTAB solution followed by chloroform extraction.

### Genotyping and SNP quality filtering

About 5000 SNP markers were assayed using the Infinium 6K Oat array (Tinker et al. 2014). Array fluorescence signals were imported and normalized in the GenomeStudio software (Illumina Inc., San Diego, CA) and then exported for the following analyses. Genotype calling in allopolyploids can be complicated, e.g., in allopolyploid wheat because of, amongst others, low signal intensity and variable hybridization of homoeologous loci (Wang et al. 2014). We used hybridization intensities as continuous scores, instead of separating the signals in clusters for genotype calling, i.e., calculating discrete variables. Continuous variables allowed us to include in the mapping population also landraces and old local varieties, which are expected to have some degree of genetic heterogeneity. For each marker, we calculated intensity ratios according to the formula X/(X + Y), where X and Y are normalized hybridization intensities for alleles A and B. Total intensity signals (*R*) and intensity ratios were used for marker and sample quality checking. Single data points with extreme *R* values (*R* < 0.25 or *R* > 2.5) were set as missing and markers with more than 25 % missing values were excluded. Variance of intensity ratios per marker was used to remove uninformative markers (variance < 0.001). After these filters, remaining missing values were replaced by the mean ratio per marker. Moreover, seven samples were removed, since the intensity ratio frequency distribution per sample indicated high levels of heterogeneity. These quality checks resulted in a dataset of 131 samples, including five diploid *A. strigosa* samples, and 3567 SNPs.

### Field trials phenotypic evaluation

The mapping population—containing 126 hexaploid samples (five diploid *A. strigosa* samples were excluded)—was divided into two sets, hereafter referred as AVEQ08 and AVEQ09 containing 75 and 62 accessions, respectively, which were evaluated in eight European locations (France, Italy, Estonia, Czech Republic, Poland, Bulgaria, Romania and Germany) either in 2008 (AVEQ08) or in 2009 (AVEQ09). Eleven accessions were included in both sets as standard cultivars. The two sets were spring-sown in randomized block experiments and were phenotyped for hull percentage and days to heading. In addition, lemma colour was evaluated in the Fiorenzuola d’Arda (Italy) field trials for both populations, using the standard 1–6 scale (white, yellow, grey, red, brown, and black).

### Growth chamber frost tolerance evaluation

The degree of leaf damage after exposure to freezing temperatures was evaluated in growth chamber experiments using chlorophyll fluorescence measurements. For each population five experiments were carried out. Seedlings were grown for 1 week at 20 °C 8 h light, 15 °C 16 h dark. First-leaf stage plants were cold-hardened for 3 or 4 weeks and then subjected to a gradual lowering of temperature for freezing treatment. Two different hardening and freezing temperatures were used, to simulate optimal and sub-optimal acclimation conditions: a 4 week acclimation period at 3 °C, 8 h light, 1 °C, 16 h dark, with a freezing treatment of 24 h at −11/−12 °C, for three out of the five experiments; and a 3 weeks period at 12 °C 8 h light, 7 °C 16 h dark, followed by a freezing treatment of 24 h at −6/−7 °C, for two experiments. After the stress period the temperature was gradually increased up to 1 °C with subsequent recovery at 20/15 °C. The experimental design was a randomized complete block design with cultivation trays as blocks (each tray containing all accessions). *F*_v_/*F*_m_ measurements were performed before, immediately after and 24 h after the stress period, using a pulse amplitude-modulated fluorometer (PAM 2000, Walz, Effeltrich, Germany), as described in Rizza et al. (2001). The measure before the stress period was used as a control.

### Data analysis

Population structure was investigated by Ward’s hierarchical clustering and Principal Component Analysis (PCA) of the marker signal ratios. Ward’s clustering was performed using the algorithm implemented in the *hclust* function of the R package *stats* (R Core Team 2014), based on a matrix of pairwise Euclidean distances. PCA was based on a correlation matrix between hybridization ratios and were conducted in R by the function *prcomp* (package *stats*). Genetic variation within and between the identified clusters was tested by Analysis of Molecular Variance (Excoffier et al. 1992) using the function *amova* in the R package *pegas* (Paradis 2010).

Phenotypic data for hull percentage and days to heading were analyzed by multifactorial analysis of variance (ANOVA), including accession and environment as fixed factors. Accession adjusted values were used to perform association mapping. Chlorophyll *F*_v_/*F*_m_ data, ranging from 0 to 0.77, were bimodally distributed with highest frequencies close to extreme values of the scale, as expected for freezing tolerance experiments that comprise susceptible damaged and tolerant non-damaged plants (Rizza et al. 2011). Thus, *F*_v_/*F*_m_ data for the 30 individual plants per accession were transformed to binomial scores using the scale mid-point (0.38) or a more extreme value to identify especially the most tolerant accession (0.6) as threshold, values above the threshold being 1 (tolerant) and below the threshold being 0 (susceptible). These 1/0 scores were subjected to logistic regression using the function *glm* of the R package *stats* (R Core Team 2014), including predictors for genotype and experiment effects. Resulting log of odds were transformed to obtain the estimated probability that each accession is tolerant to frost (hereafter called frost tolerance scores), which were used for GWAS. *F*_v_/*F*_m_ data from optimal and sub-optimal acclimation experiments were analysed both separately and together, thus estimating also an overall probability to tolerate different conditions.

Genome-wide association analyses were performed using and comparing alternative models, all fitted in GenStat (VSN International 2014). We could not use commonly available software, such as TASSEL (Bradbury et al. 2007) or EMMAX (Kang et al. 2010), because they require discrete genotypic data as input. However, the statistical approach we used is identical, namely testing the significance of marker effect in generalized linear models or linear mixed models, which correct for population structure or kinship or both. In a first step, a simple linear model was used that did not correct for population structure: y = marker + error, where y is the trait of interest, and marker is fixed. A second linear mixed model corrected for kinship between genotypes, using a variance component approach similar to the method implemented in EMMAX. The following model was used to test marker effects on each trait: y = marker + genotype + error, where genotype is a random effect and marker is fixed as before. The covariance between the different genotypes was structured by a kinship matrix, calculated as Euclidean distances based on marker scores. Different subset of equally spaced markers (160, 302 and 520 SNPs) were used for calculating the kinship matrix and their ability to correct for stratification was compared to a kinship matrix obtained using all the markers. A third fixed effects model corrected for population structure using the scores of the first *n* principal components calculated using all markers: ${\text{y}} = \mathop \sum \limits_{i} {\text{PC}}_{i} + {\text{marker}} + {\text{error}}$, where marker and vectors of principal component scores were all fixed. Models including 10, 15, 20 and 40 principal components were compared. This model uses the same approach of the method implemented in EIGENSTRAT (Price et al. 2006). A fourth linear mixed model corrected for population structure including principal components and markers as fixed factors and a kinship matrix as random, similar to the mixed linear model (MLM) included in the software package TASSEL. Resulting *p* values from the association analyses were plotted versus the expected *p* values, which are assumed to be uniformly distributed, under the null hypothesis.

A genome-wide significance threshold was calculated by the method described in Li and Ji (2005) for AVEQ08, AVEQ09 and for the whole dataset. Genome-wide p values, indicating the strength of the genotype–phenotype association, were visualized as Manhattan plots using SNP map positions reported by Chaffin et al. (2016).

After the association analysis, intensity ratio frequency distributions of significantly associated markers were visually checked to exclude association due to a few outliers. This check has the same aim of filtering out markers with a low minor allele frequency (MAF) before association analysis, as is usual in standard approaches using discrete genotypes.

Analysis of Linkage Disequilibrium (LD) decay as function of the pairwise distance between markers was conducted using the function *LD.Measures* from the R package *LDcorSV*, correcting for population stratification using a covariance matrix between individuals (Desrousseaux et al. 2013). The pairwise marker correlations (*R* squared) within chromosomes were calculated and grouped in 1 cM large bins. Then, the 90th percentiles of the *R* squared distributions per bin were smoothed by a spline function.

## Results

### Population structure

**Fig. 1:**
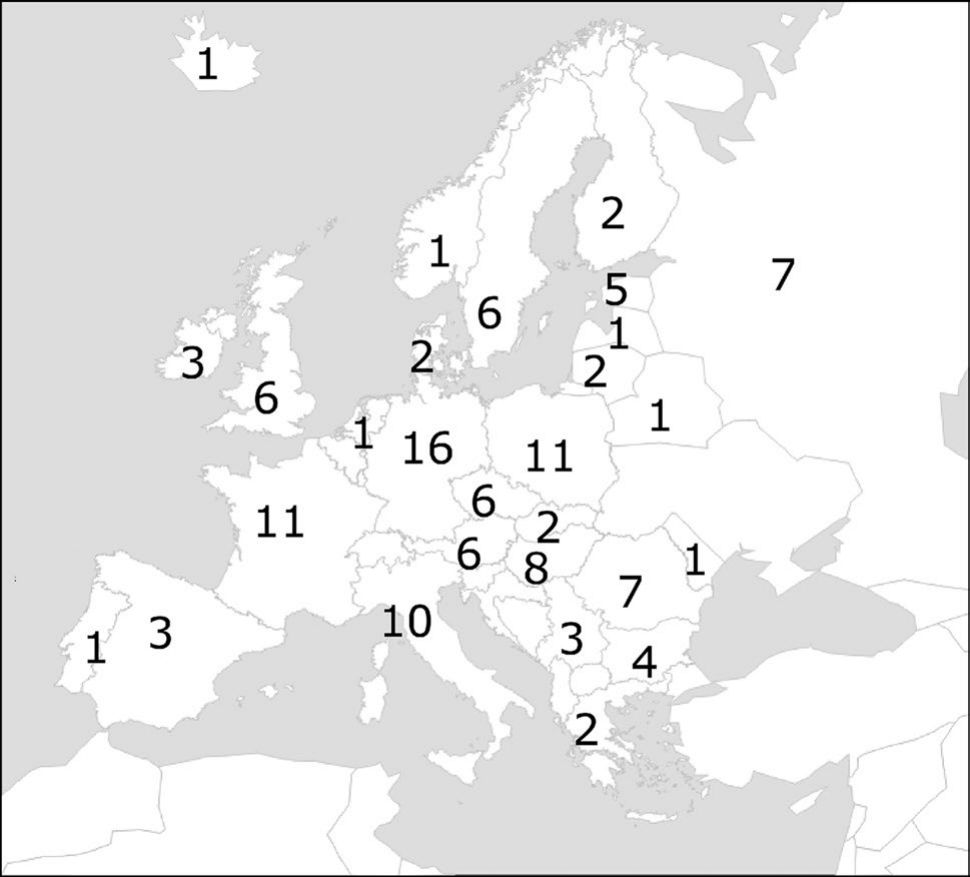
Map of Europe showing the composition of the oat population in terms of country of origin. The number of accessions per origin country is indicated

**Fig. 2:**
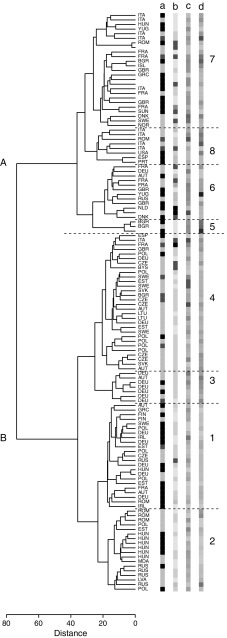
Ward’s dendrogram based on genotypic pairwise Euclidean distances. *Labels* indicate origin countries of the samples and colour gradients are relative to (*a*) mapping population composition (*grey* for AVEQ08, *black* for AVEQ09, *red* for standard cultivars), (*b*) lemma colour accession means, (*c*) frost tolerance scores (from *blue* for frost tolerant to *red* for frost susceptible) and (*d*) days to heading accession adjusted values (from *blue* for late-flowering accessions to *red* for early-flowering accessions). To visualize the phenotypic variation across the whole population, the year effects for frost tolerance scores and days to heading accession adjusted values were compensated using the eleven standard cultivars present in both populations

**Fig. 3:**
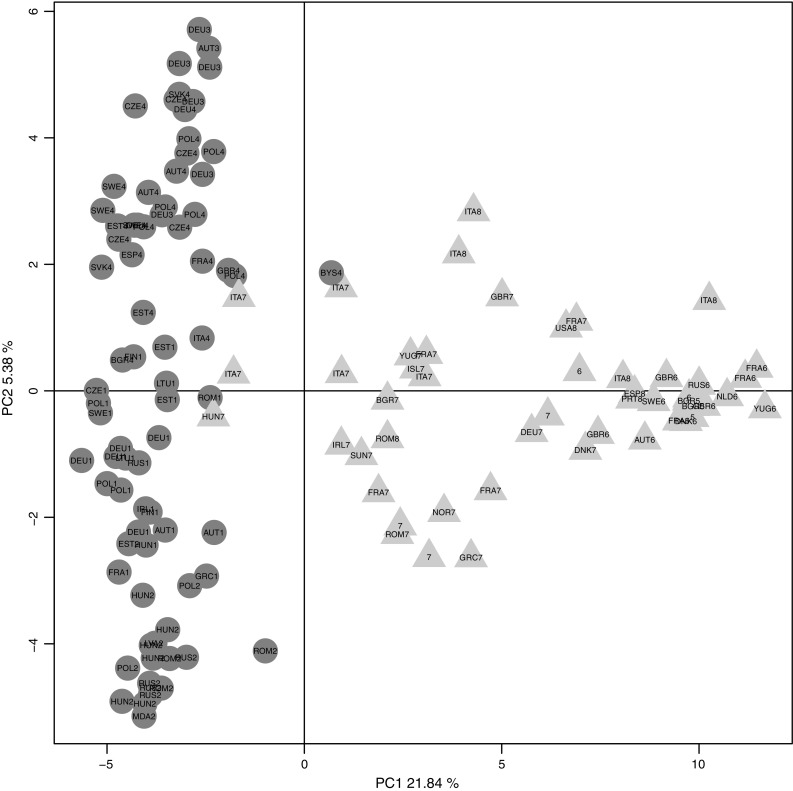
Principal component analysis based on 3567 SNP hybridization intensity ratios. A *scatter plot* of PC1 (explaining 21.84 % of the variance) versus PC2 (explaining 5.38 % of the variance). *Labels* indicate origin countries of the samples and Ward cluster assignments. *Colours* and *symbols* according to the two main groups defined by Ward’s clustering (group A, *blue triangle*; group B, *orange circle*)

In this study, hybridization intensities from the Illumina 6K Oat SNP array were used to determine population structure in a collection of 131 European oat accessions (Fig. 1). Five diploid *A. strigosa* accessions were included in a first PCA and then removed from the following analyses, as they clearly clustered far from hexaploid oat samples (data not shown). Ward’s hierarchical clustering revealed the presence of two main groups, which can be further subdivided giving a total of eight subgroups. In Fig. 2, the Ward’s dendrogram is presented together with accession origin (country) and colour scales according to the phenotypic scores for lemma colour, frost tolerance, and days to heading. Analysis of principal components was first conducted on the whole population (Fig. 3) and then, as a nested analysis, on each of the two main groups (Online Resource 2). The pattern of clusterization obtained by PCA was in good accordance with Ward’s clustering, supporting the dendrogram morphology (Fig. 3, Online Resource 2). Population structure analysis indicated a clustering related to geographical origin of the accessions, separating Mediterranean and Atlantic European accessions (group A) from Continental European accessions (group B) (Figs. 2, 3). Mediterranean and Atlantic European accessions are distinguished from Continental European accessions by PC1, while PC2 shows variability among Continental European accessions. Within the Continental European group, subgroup 2 contains accessions from Eastern countries (Hungary, Romania and Russia), subgroup 3 from Germany, subgroups 1 and 4 mainly from Central and Northern Europe (Austria, Czech Republic, Germany, Poland, and Sweden) (Figs. 2, 3). As for group A, subgroup 5 consists of three accessions from Bulgaria, subgroup 6 mainly from Great Britain and France, and subgroup 8 from Italy and Spain (Figs. 2, 3).

A two-level Analysis of Molecular Variance (AMOVA) found a significant difference between the Mediterranean/Atlantic Europe (A) and the Continental Europe (B) groups, explaining 21.9 % of the total variance (Table 1). Molecular variance within group A (152.8) was higher than within group B (81.2), in agreement with higher variability observed in group A for lemma colour, frost tolerance and heading date (Fig. 2). AMOVA also revealed significant differences among subgroups, accounting for 18.4 % of the total variance, with the largest part of variance (59.7 %) remaining within subgroups.

**Table 1:** Two-level analysis of molecular variance (AMOVA), showing significant differences between the Mediterranean/Atlantic Europe (A) and the Continental Europe (B)

Variance components	Variance	% Total	*p*	Φ-Statistics
Between groups (σ_a_^2^)	33.321	21.90	<0.0001	Φ_CT_ = 0.219
Among subgroups (σ_b_^2^)	28.017	18.41	<0.0001	Φ_SC_ = 0.236
Within subgroups (σ_c_^2^)	90.842	59.69		Φ_ST_ = 0.403

### Phenotypic variation

The phenotypic data for hullessness, lemma colour, days to heading and frost tolerance were scored in field trials or in growth chambers in 2008 and 2009. As only few accessions were present in both years, we decided to analyse the data for the accessions of the 2 years separately, hereafter called the AVEQ08 and AVEQ09 datasets.

Naked (or hulless) oats were represented by six accessions in AVEQ08 and three accessions in AVEQ09, two of them in common to both groups as standard cultivars. The accession adjusted values for hull percentage were clearly distributed into two classes, in the range 6–18 % for naked and multiflorous oats and 36–62 % for covered oats (Online Resource 3).

Lemma colour scores of the populations ranged from 1 to 5, with a similar frequency distribution. The pigmented categories (grey, red and brown) together represented 20 % of the total number of accessions (Online Resource 3).

Days to heading was in the range 56–82 for AVEQ08 and 60–92 for AVEQ09 (Online Resource 3).

Chlorophyll fluorescence measurements were used to evaluate frost tolerance. The AVEQ08 and AVEQ09 accessions were separately phenotyped in five experiments per set. Frost tolerance scores ranged from 0.11 to 1 in AVEQ08 and from 0.07 to 0.98 in AVEQ09. Logistic regression provided a good description of the data for both populations and accession effects were highly significant (*p* ≤ 0.0001). Frequency distribution plots of frost tolerance scores can be found in Online Resource 3. Residual plots were performed and no substantial deviations were observed from the assumption of normally distributed residuals with constant variance.

### Correlation between structure and phenotypic variation

Almost all accessions of group B had white or yellow lemmas, only 6 % was red or black oat. The accessions in group A appeared to be more diverse with 26 % red/brown oat, 17 % grey oat and only 52 % white/yellow oat (Fig. 2). Frost tolerance scores of the accessions correlated with population structure (Fig. 2). The most frosttolerant accessions belonged to the Mediterranean and Atlantic European group and, in particular, were grouped in three branches in subgroups 5, 6 and 7 (Fig. 2). In contrast, only a few moderately tolerant accessions were present in group B (gathered in subgroup 2), while subgroups 3 and 4 consisted entirely of accessions that were susceptible to frost. Heading date correlated with clustering as well (Fig. 2). The group B appeared quite homogeneous and was dominated by early-flowering or intermediate accessions, while group A showed large variation in flowering date, with some very early-flowering accessions in clusters 8 and 7 (especially from Italy) and late-flowering accessions in clusters 5, 6 and 7, most of which were frost-tolerant.

### Correction for population stratification

**Fig. 4:**
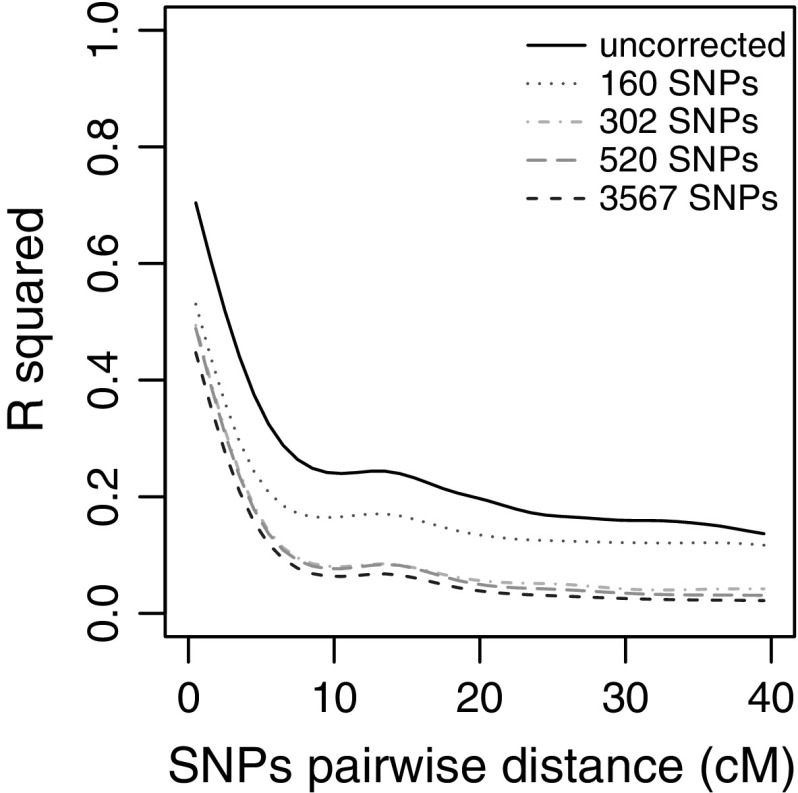
Decay of linkage disequilibrium as function of the pairwise SNP distance

The GWAS results presented below were obtained using the model including a kinship matrix based on 302 uniformly spaced markers, since in most of the analyses it outperformed the other models in controlling false positives (Online Resource 7). The choice of this kinship matrix was also supported by the analysis of LD decay, indicating that the subset of 302 markers contains most of the genetic information; by adding more markers, only a slight improvement of LD correction is obtained (Fig. 4). The genome-wide significance thresholds (with α = 0.05) were 3.365, 3.246 and 3.551 (−log*P*) for AVEQ08, AVEQ09 and the whole dataset, respectively.

### Genome-wide association analyses for simple traits: hullessness and lemma colour

**Fig. 5:**
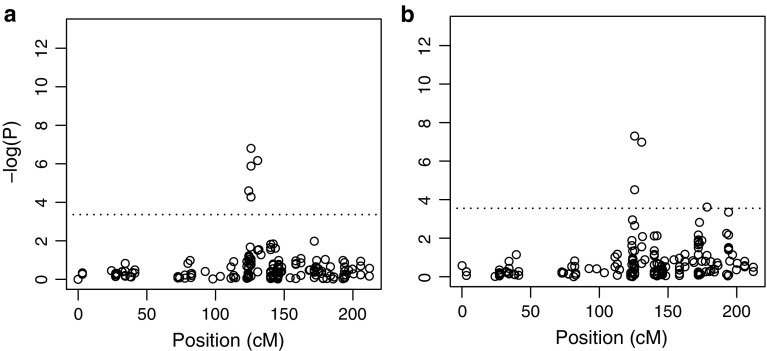
Manhattan plot of −log*P* values calculated by GWAS for hull percentage in AVEQ08 (**a**) and for the qualitative naked status in the whole collection (**b**). The linkage group Mrg21 is shown with marker genetic position relative to the most recent consensus map (Chaffin et al. 2016). The *horizontal dotted line* represents the genome-wide significance threshold

Association analyses for lemma colour and hullessness were used for testing the effectiveness of our approach in detecting association for simple traits, whose phenotypic variation is probably explained by few major genes. Two strong associations were found for hull percentage in AVEQ08 in the linkage groups Mrg21 (five markers mapping from 123.7 to 130.8 cM with the maximum −log*P* higher than 7) and Mrg04 (seven markers co-mapping at 45.6 cM with −log*P* ranging from 7 to 9) (Fig. 5 and Online Resource 4). Other two markers were significantly associated to the trait in Mrg09 and Mrg03 (−log*P* > 5 and −log*P* > 4, respectively). Moreover, four markers with unknown map position were significantly associated (Online Resource 4). Although phenotypic variation in AVEQ09 was low (only three naked oats) four regions were found significantly associated and three of them co-localized with the QTLs found in AVEQ08 on Mrg21, Mrg04 and Mrg09 (Online Resource 4). By mapping the naked condition (or multiflorous spikelet, which is genetically linked) as a qualitative trait across the whole population (AVEQ08 plus AVEQ09), the two QTLs in Mrg21 and Mrg04 were confirmed and two other markers were found significant in Mrg21 (at 178.3 cM) and Mrg08 (at 132.4 cM) (Fig. 5 and Online Resource 4).

GWAS for lemma color resulted in two robust associations with the markers GMI_ES15_c2369_181 in linkage group Mrg20 (14.7 cM) and GMI_ES_LB_8315 with unknown position, as they were significant for both mapping populations (Online Resource 5). Two other putative QTLs in linkage groups Mrg03 (56.2 cM) and Mrg17 (84.3 cM) were detected in AVEQ08 and four QTLs in Mrg02 (27.4), Mrg03 (5.1 cM), Mrg04 (32.7 cM) and Mrg12 (35.3 cM) in AVEQ09 (Online Resource 5). Eleven significant markers for AVEQ09 were excluded because of the presence of outliers.

### Genome-wide association analyses for complex traits: frost tolerance and heading date

**Fig. 6:**
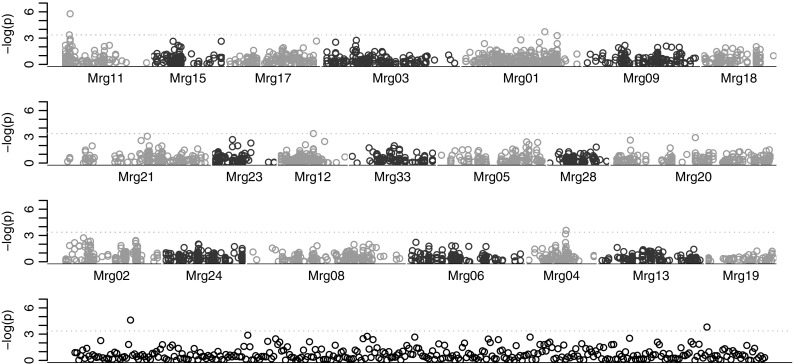
Manhattan plots of −log*P* values calculated by GWAS for frost tolerance in AVEQ08. Genetic position of markers is relative to the most recently available consensus map by Chaffin et al. (2016). *Black points* indicate unmapped markers. The *horizontal dotted line* represents the genome-wide significance threshold

Association analysis for frost tolerance detected six significant markers, three for AVEQ08 in Mrg01 (101.5 cM) and Mrg11 (8.8–9.8 cM) and three for AVEQ09 in Mrg12 (58.5 cM), Mrg20 (156.7 cM) and Mrg21 (205.7 cM) (Table 2). The −log*P* values for marker effects for AVEQ08 are shown in Fig. 6 as a Manhattan plot. Four significant markers detected for AVEQ08 (in Mrg04, Mrg12 and two unknown positions) were excluded because of the presence of outliers (Fig. 6). The marker GMI_ES05_c13603_259 mapping in Mrg11 (9.8 cM) showed the most significant effect, with −log*P* greater than six. Association mapping results were not very sensitive to the threshold used for the binomial transformation of *F*_v_/*F*_m_ values (data not shown). If separately analysed, frost tolerance scores from the two hardening conditions (optimal and sub-optimal hardening) were associated to different regions (Table 2). No markers were detected for frost tolerance scores measured after sub-optimal hardening for AVEQ09.

**Table 2:** List of associated markers for frost tolerance in AVEQ08 and AVEQ09

Locus_Name	−log*P*	Group	Position	Chrom
AVEQ08 overall				
GMI_ES01_c1416_473	3.72	Mrg01	101.5	5C
GMI_ES01_c30278_396	3.38	Mrg11	8.8	1C
GMI_ES05_c13603_259	5.77	Mrg11	9.8	1C
AVEQ09 overall				
GMI_DS_LB_1269	3.27	Mrg12	58.5	13A
GMI_ES01_c26788_88	3.39	Mrg20	156.7	19A
GMI_GBS_67251	3.59	Mrg21	205.7	8A
AVEQ08 sub-opt				
GMI_DS_CC7686_215	3.45	Mrg03	58.6	4C
GMI_ES02_c911_580	3.50	Mrg15	87.2	2C
GMI_ES01_c10257_104	4.10	Mrg23	28.5	11A
GMI_ES15_c6451_437	3.84	Mrg23	28.5	11A
GMI_GBS_17527	3.70	Mrg33	26.3	15A
AVEQ08 optimal				
GMI_DS_CC6027_225	4.28	Mrg02	27.4	9D
GMI_ES15_c276_702	3.56	Mrg11	3.7	1C
GMI_ES05_c13603_259	4.25	Mrg11	9.8	1C
GMI_ES_CC16445_119	3.54	Mrg17	114.5	3C
GMI_ES_CC11076_204	3.66	Mrg20	97.1	19A
AVEQ09 optimal				
GMI_ES02_c3577_672	3.87	Mrg01	117.4	5C
GMI_DS_LB_7011	3.49	Mrg08	142	12D
GMI_GBS_67251	3.76	Mrg21	205.7	8A
GMI_DS_LB_6024	3.51	Mrg28	54.2	17A
GMI_ES02_c27548_253	3.86	NA	NA	NA

Regarding days to heading, in total seven markers were significant, but one of them with unknown map position was excluded due to the presence of outliers. Significant associations were found in Mrg01 (117.9 cM), Mrg02 (33 cM) and Mrg13 (58.6 cM) for AVEQ08 and in Mrg20 (18 and 115.6 cM) and Mrg21 (122.8 cM) for AVEQ09 (Online Resource 6).

## Discussion

### SNP signals analysis

In oat genetic diversity studies, dominant and co-dominant markers such as AFLP, SSR and DArT have been used to analyse DNA samples extracted from bulks of several individuals, to take into account the within-accession genetic variability (Fu et al. 2005; Achleitner et al. 2008; Tinker et al. 2009; Montilla-Bascon et al. 2013). Especially when within-accession variability is expected to be large, as in the case of landraces, bulking more plants allows sampling genetic variability while limiting genotyping costs.

In the present work, DNA samples from bulks of 10 plants were analysed using an Infinium SNP array. We genotyped a collection of European oat accessions obtained from genebanks, including modern and old varieties as well as a few landraces, for which a certain degree of genetic heterogeneity was expected. Due to a high signal to noise ratio, SNP arrays enable accurate estimation of allelic ratios in polyploids (e.g., Bertioli et al. 2014, using a GoldenGate array in tetraploid peanut; Bourke et al. 2015, using an Infinium array in tetraploid potato; Bassil et al. 2015, using an Axiom array in octaploid strawberry). To what extent they are able to detect low frequency alleles in bulks of samples has not been quantified. We used SNP hybridization intensity ratios as continuous variables representing accession-level (bulk) allele frequencies. Similar to genotypes for individuals, bulk allele frequencies can be statistically associated to phenotypes measured in bulks in GWAS. For instance, Montilla-Bascon et al. (2015) performed a GWAS for crown rust and powdery mildew using SNP data of bulks of oat varieties and landraces. Bulking enabled us to reduce genotyping costs. Moreover, using intensity ratios, SNP signals were not interpreted as discrete genotype classes, reducing the risk of loss useful information for genetic diversity analysis and GWAS (Miller et al. 2013; Myles et al. 2015). This appears particularly relevant for allopolyploid species, as SNP probes may or may not be subgenome-specific (Bassil et al. 2015), making genotype calling a challenging, time-consuming and error-prone task.

In association studies, the presence of population structure can be a source of false positives and a statistical correction is usually included in the GWAS model. We used several models which correct for population stratification in different ways: (1) including principal components as fixed cofactors, similar to the approach implemented in EIGENSTRAT (Price et al. 2006); (2) using a mixed model including a kinship matrix, as also implemented in e.g., TASSEL (Bradbury et al. 2007) or EMMAX (Kang et al. 2010); (3) including in a mixed model principal components as fixed factors and a kinship matrix as a random factor, implemented in TASSEL as well. Unfortunately, it was not possible to use standard software packages commonly used for GWAS because they require discrete genotypes as input. While the approach we used can be considered equivalent to those most commonly used, the calculation of the kinship matrix was slightly different. We point out that, when the genetic data are continuous rather than discrete, our approach calculating kinship as Euclidean distances is more appropriate than methods commonly applied to discrete genotypes.

Our association model including a kinship matrix, based on 302 equally spaced markers, considerably reduced the deviation of the observed *p* values frequency distribution from the assumption of uniformity, notably for frost tolerance, heading date and lemma color (Online Resource 7).

### Population structure

Principal components analysis and Ward’s clustering clearly revealed the presence of population structure, mainly related to the geographical origin of the accessions, which is in agreement with several studies of large oat germplasm collections using various genotyping methods (Fu et al. 2005; Tinker et al. 2009; Huang et al. 2014; Tinker et al. 2014). Oat breeding programs appear to have a tendency to use their own lines with limited introgression of plant materials from other countries.

The two main groups identified in the present work (Mediterranean/Atlantic Europe and Continental Europe) may be related to growth habit (spring vs. winter type). Indeed, although growth habit classification was not available for all accessions, the most frost-tolerant accessions (as defined in this work by *F*_v_/*F*_m_ measurements) clustered only in group A from Mediterranean/Atlantic Europe, where fall-sowing is permitted by milder winter temperatures. In particular, they mainly clustered into two branches of the Ward’s dendrogram in subgroups 6 and 7, and the available pedigrees lead to the ancestors Winter Turf (also called Grey winter) and Grise d’hiver, two European winter landraces with many similar features (Stanton 1955; Hunter and Carson 1949). The SNP array we used has been shown to correctly predict the growth habit in a diverse collection of 595 accessions from USA, Canada, Brazil and Europe (Tinker et al. 2014), but in contrast to that study we did not obtain a single cluster of winter types (or frost-tolerant types). This could have several possible explanations: (1) the winter types analysed by Tinker et al. (2014) were mainly from USA and this may have underestimated the diversity of worldwide winter types; (2) admixture may have taken place in the European material, so that the modern varieties included in our study do not strictly reflect any more a stratification related to growth habit.

The taxonomical distinction that used to be made between *Avena sativa* L. (common oat) and *Avena sativa* spp. *byzantina* K. Koch (red oat) is another source of stratification detected by many genetic diversity studies including hexaploid oat (Fu et al. 2005; Achleitner et al. 2008; Montilla-Bascon et al. 2013; Newell et al. 2011), which may suggest the existence of two significantly differentiated gene pools. Based on the mode of floret separation (Diederichsen 2007), the only red oat included in our population is the Italian variety named Argentina (subgroup 8). Consistent with previous studies on the distance between common and red oat, the variety Argentina was genetically the most distinct accession. In the PCA of group A, Argentina had an extreme position (data not shown). Subgroup 8 was highly diverse, as indicated by the highest within-group Mean Sum of Squares (178).

### Genome-wide association analyses for simple traits

We performed GWAS using hybridization ratios from 3567 SNP markers. This surpassed the minimum number of 2000 markers (one marker every cM) proposed for genome-wide association studies in oat, based on the analysis of LD in a large set of 1205 oat lines (Newell et al. 2011). Although the number of markers we used suggests a good genome coverage, we cannot exclude the presence of gaps, since for about 1000 markers the genetic map position is currently unknown. A higher number of markers would increase the average level of linkage between markers and QTLs and therefore the power of QTL detection.

According to the currently accepted genetic model for hullessness, the incompletely dominant *N1* gene expression is modulated by genetic background (Ougham et al. 1996). The N1 locus was previously identified in linkage group TM-5 (Terra x Marion) (De Koeyer et al. 2004) which is homologous to linkage group Mrg21. Using hull percentage, we found two robust associations in linkage groups Mrg21 and Mrg04 (around 125 and at 45.6 cM, respectively; Fig. 5 and Online Resource 4) and this finding was consistent across the two mapping populations. By mapping the naked status (strictly linked to multiflorous spikelet) as a qualitative trait, an additional significant association was found in Mrg21 at 178.3 cM (Fig. 5), mapping closer to the marker cdo482 (199.2 cM) reported by De Koeyer et al. (2004) as associated to locus N1 in the bi-parental population Terra x Marion. De Koeyer et al. (2004) also found an additional association using a quantitative score for hullessness (hull percentage), but unfortunately that marker was not linked to any linkage group.

The association we found for lemma colour with the marker GMI_ES15_c2369_181 in linkage group Mrg20 (14.7 cM) has never been identified earlier. However, this association showed a very significant *p* value (−log*P* > 5 in AVEQ08 and −log*P* > 7 in AVEQ09) and most importantly was consistent across the two mapping populations, supporting our finding. These results showed that our GWAS approach based on SNP array intensity ratios was effective to detect significant associations for simply inherited traits.

### Genome-wide association analyses for frost tolerance and heading date

Significant associations for frost tolerance were found in linkage groups Mrg01 (101.5 cM) and Mrg11 (9.8 cM) for population AVEQ08 and in linkage groups Mrg12 (58.5), Mrg20 (156.7 cM) and Mrg21 (205.7) for AVEQ09 (Table 2).

Previous studies have identified several QTLs related to frost tolerance or winter hardiness. Nava et al. (2012) cloned the homologous genes for *Vrn*-*1* and *Ft*-*1* (*Vrn*-*3*). *Vrn1*-*T4* showed the highest homology to the wheat gene *Vrn*-*A1.* In the Kanota x Ogle map (KO; facultative x spring type) (Wight et al. 2003), *Vrn1*-*T4* co-located with a known QTLs affecting flowering time and crown freezing tolerance on linkage group 24_26_34 (Nava et al. 2012; Tinker et al. 2009; Holland et al. 2002; Wooten et al. 2008). This QTL including the *Vrn*-*1* locus is located in a region of about 10 cM from 130 to 140 cM in Mrg20 of the consensus map, which is very close to the main association we detected in Mrg20 at 143.6 cM for AVEQ09. However, sequence alignment and map homology indicated that a second copy of *Vrn*-*1* may be located on the homoeologous KO group 22_44_18 (Nava et al. 2012), corresponding to Mrg21. These two loci could represent the associations we found for AVEQ09 in Mrg20 (156.7 cM), which maps 15 cM apart from *Vrn*-*1,* and Mrg21 (at 205.7 cM).

The locus *Fr*-*2*, affecting frost tolerance in *Triticeae*, has not been identified yet in oat. Although one of the QTLs we found could potentially represent *Fr*-*2*, currently we do not have enough data to discuss it. According to the 6 K SNP array annotation (Tinker et al. 2014), the significant marker we found in Mrg01 (101.5 cM) for AVEQ08 is located in the reciprocal genomic translocation 17A-7C. This region has been previously associated to winter survival and crown freezing tolerance tracking this translocation by FISH in the population Wintok x Fulghum (Santos et al. 2006) and using marker-based QTL mapping in the population Fulghum x Norline (Maloney et al. 2011), although its effect on frost tolerance was not confirmed in the KO population (Wooten et al. 2008). Unfortunately, due to the low resolution of the Fulghum x Norline map it is not possible to make a precise comparison of the QTL position.

Using the KO population Wooten et al. (2008) identified seven QTLs for crown freezing tolerance, among which the QTL with the largest effect mapped on KO 24_26_34, most probably detecting the *Vrn*-*1* locus (Mrg20) described above. Six other QTLs with minor effects were located in KO linkage groups 21 + 46_31 + 4, 16_23, 25, 3 + 38, 11_41 + 20 and 22_44_18. The QTL found in KO 21 + 46_31 + 4, and in particular marker BCD1230B, maps in Mrg11 at 40.8 cM in the new consensus map. The most robust association we found in Mrg11 maps at 9.8 cM, which therefore could be considered a new QTL.

The two hardening condition had an effect on association mapping as different QTLs were detected, suggesting that the accessions interact differently with the different hardening conditions. Significant associations for frost tolerance in Mrg23 (28.5 cM) and Mrg33 (26.3 cM) were detected only for sub-optimal acclimation condition experiments (Table 2) and were not found in earlier studies. The main QTLs in Mrg11, Mrg01, Mrg20 and Mrg21 were detected both using the optimal hardening condition and using the overall frost tolerance scores (Table 2).

The differences in QTLs found between the two datasets analysed here may partly be related to the difference in genetic structure (Fig. 2a). AVEQ08 was well-balanced, representing all the genetic subgroups identified by Ward’s clustering and PCA. AVEQ09 was narrower in terms of subgroups and also had a smaller size, so a lower statistical power may be expected.

#### Author contribution statement

VT and CUG conceived the study. VT planned and supervised the genotyping work. CUG coordinated the AVEQ project in which FR and FWB carried out the growth chamber experiments for frost tolerance evaluation. CM and RG developed the mapping populations, extracted the DNA samples and contributed to the frost tolerance evaluation. GT performed the statistical analyses of phenotypic data, genetic diversity, and genome-wide association and prepared the manuscript. REV contributed to SNP data curation and association analysis. MJP provided statistical support for genome-wide association analyses. REV and MJMS supervised all the data analyses and contributed to the interpretation of the data. REV, MJMS, MJP, FR, FWB and VT commented and revised the manuscript.

## Electronic supplementary material

Below is the link to the electronic supplementary material.

**OR01.** List of accessions analysed in the present study, with additional information on accession name, origin country, annuality, registration date (PDF 77 kb)

**OR02.** PCA plots based on 3567 SNPs for the two main clusters detected by Ward’s method: (a) group A and (b) group B. Accessions are represented by numbers indicating Ward subgroup assignments (PDF 32 kb)

**OR03.** Frequency distribution of the phenotypic variables used for GWAS. Lemma colour accession means (c), accession adjusted values for hull percentage (a) and heading date (e) and frost tolerance scores (g) in AVEQ08 and AVEQ09 (d, b, f, h, respectively) are shown (PDF 6 kb)

**OR04.** Manhattan plots of –log *P* values (ordered by chromosome) calculated by GWAS for hull percentage in AVEQ08 (a) and AVEQ09 (b). Genetic position of markers is relative to the most recently available consensus map for oat (Chaffin et al. 2016). Black points indicate unmapped markers. The horizontal dotted line represents the genome-wide significance threshold (PDF 44 kb)

**OR05.** List of associated markers for lemma color in AVEQ08 and AVEQ09 (DOCX 15 kb)

**OR06.** List of associated markers for heading date in AVEQ08 and AVEQ09 (DOCX 12 kb)

**OR07**. GWAS observed *p* -values versus expected *p* -values (Quantile–quantile plot) for frost tolerance (a for AVEQ08 and b for AVEQ09), heading date (c for AVEQ08 and d for AVEQ09), lemma colour (e for AVEQ08 and f for AVEQ09), and hull percentage (g for AVEQ08 and h for AVEQ09). The red line represents the *p* -values distribution for simple association and the blue line represents the *p* -values for the model that corrects for kinship using a subset of 302 uniformly spaced markers (PDF 365 kb)
