# Supplementary material for: Population structure and genome-wide association analysis for frost tolerance in oat using continuous SNP array signal intensity ratios
Source: Theor Appl Genet. 2016 Jun 18;129:1711–24. doi: 10.1007/s00122-016-2734-y (PMC4983288; doi:10.1007/s00122-016-2734-y)
Supplement: Supplementary file 1 — OR01. List of accessions analysed in the present study, with additional information on accession name, origin country, annuality, registration date (PDF 77 kb) [file 122_2016_2734_MOESM1_ESM.pdf]

| FAO.code | Accession.number | Species | Country | Accession.name | Registration | Annuality |
|---|---|---|---|---|---|---|
| AUT001 | BVAL-450001 | sativa | AUT | Attergauer Nackthafer | 1950 | |
| AUT001 | BVAL-451001 | strigosa | AUT | Sandhafer | 1976 | |
| AUT005 | RINN-455018 | sativa | AUT | Spitzhafer | | |
| AUT016 | Cavallo | sativa | AUT | Cavallo | 2001 | Spring |
| AUT016 | CPVO19952388 | sativa | AUT | MONARCH | 1996 | Spring |
| AUT056 | CPVO20062595 | sativa | AUT | EFFEKTIV | 2006 | Spring |
| BGR001 | A7BM0001 | sativa | BGR | Mina | | Spring |
| BGR001 | A7BM0002 | sativa | BGR | Dulo | | Winter |
| BGR001 | A7BM0005 | sativa | BGR | 83/200-CR | | Winter |
| BGR001 | A7BM0006 | sativa | | | | |
| BGR001 | BGR 12088 | sativa | FRA | AVOINE DU PRIEURE | 1982 | |
| BGR001 | BGR 24958 | sativa | | WORONEZH | 1983 | |
| BGR001 | BGR 24983 | sativa | BGR | K 06-08 | | Spring |
| BGR001 | BGR 25059 | sativa | | | | |
| BGR001 | BGR 7982 | strigosa | | | 1939 | |
| BOREAL | VELI | sativa | FIN | VELI | | |
| CZE047 | 03C0700014 | sativa | CZE | Brnensky Zlatak | 1936 | Spring |
| CZE047 | 03C0700106 | sativa | YUG | Z 164 | 1974 | Spring |
| CZE047 | 03C0700402 | sativa | HUN | Szegedi | 1964 | Winter |
| CZE047 | 03C0700819 | byzantina | ITA | Puglieze | 1952 | Spring |
| CZE047 | 03C0701326 | sativa | SUN | Uzbeckij Sirokolistyj | 1980 | Spring |
| CZE047 | 03C0701732 | sativa | CZE | Auron | 1991 | |
| CZE047 | 03C0701982 | sativa | CZE | Dalimil | | |
| CZE047 | Saul | sativa | CZE | Saul | 2005 | |
| CZE074 | Raven | sativa | CZE | Raven | 2008 | |
| DEU060 | Flaemingsgold | sativa | DEU | Flämingsgold | 2008 | Spring |
| DEU060 | LPSH 02-202 | sativa | DEU | LPSH 02-202 | | Spring |
| DEU060 | LPSH 02-239 | sativa | DEU | LPSH 02-239 | | Spring |
| DEU087 | CPVO20040091 | sativa | DEU | IVORY | 2004 | Spring |
| DEU087 | CPVO20060096 | sativa | DEU | TYPHON | 2006 | Spring |
| DEU087 | CPVO20070095 | sativa | DEU | PERGAMON | 2007 | Spring |
| DEU087 | CPVO20080196 | sativa | DEU | Scorpion | 2008 | Spring |
| DEU146 | AVE 1284 | sativa | ROM | | | |
| DEU146 | AVE 271 | sativa | GRC | | | |
| DEU146 | AVE 313 | sativa | DEU | Rotenburger Schwarzhafer | 1922 | |

| | | | | | | |
|---|---|---|---|---|---|---|
| DEU146 | AVE 3332 | sativa | DEU | Nuernberg 8 ("100 Jaehriger Hafer") | 1832 | Spring |
| DEU146 | AVE 3334 | sativa | DEU | Weihenstephaner Weiss | | Spring |
| DEU146 | AVE 35 | strigosa | | Rauhhafer aus Hamstedt | | |
| DEU146 | AVE 399 | sativa | DEU | Rheinischer Gelb | 1933 | |
| DEU146 | AVE 4388 | sativa | DEU | Borreck | 1960 | Spring |
| DEU146 | AVE 4659 | sativa | DEU | Lueneburger Kley Neue Zucht | 1950 | Spring |
| DEU146 | AVE 615 | sativa | DEU | Gelber Riesenfahnen | 1891 | |
| DEU387 | CPVO20040125 | sativa | DEU | KAPLAN | 2004 | Spring |
| DEU481 | CPVO20010181 | sativa | DEU | LEO | 2001 | Spring |
| ESP004 | BGE002046 | sativa | ESP | Avena | 1979 | |
| ESP004 | BGE004766 | strigosa | ESP | Avea negra | 1981 | |
| ESP004 | BGE025428 | sativa | ESP | Avena de boruca | 1996 | |
| EST001 | 00004 | sativa | EST | Jaak | 1995 | |
| EST001 | 00005 | sativa | EST | Jõgeva Agu | 1939 | |
| EST001 | 00011 | sativa | EST | Miku | 1991 | |
| EST001 | 00012 | sativa | EST | Viker | 1976 | |
| EST001 | 00013 | sativa | EST | Villu | 1999 | |
| FRA040 | 19283 | sativa | FRA | NOIRE DE L'AUBE | 1954 | Spring |
| FRA040 | 19303 | sativa | FRA | NUE GROSSE | 1978 | Spring |
| FRA040 | 19357 | sativa | FRA | GRISE DE HOUDAN | 1912 | Spring |
| FRA040 | 19371 | sativa | FRA | JOANETTE (AVOINE DE CHENAILLES) | 1888 | Spring |
| FRA040 | 19494 | sativa | | SAINT PAUL LE FROID | 2000 | |
| FRA040 | 19619 | sativa | FRA | BLANCHE NEIGE | 1956 | Winter |
| FRA040 | 19620 | sativa | FRA | LA GAILLARDE | 1950 | |
| FRA040 | 19631 | sativa | FRA | SOUVERAINE LEPEUPLE | 1940 | Winter |
| FRA040 | 19632 | sativa | USA | CIMARRON | 1954 | Winter |
| FRA040 | 30040 | sativa | FRA | AUTEUIL | | Spring |
| FRA261 | AINTREE | sativa | FRA | AINTREE | 1992 | Winter |
| FRA261 | CPVO19981528 | sativa | FRA | EVORA | 1999 | Winter |
| GBR011 | 00004 | sativa | GBR | FELTWELL | 1964 | Winter |
| GBR011 | 00009 | sativa | GBR | PENRHYN | 1958 | Winter |
| GBR011 | 00036 | sativa | YUG | BELYSKI (BELJSKA 200) | | Winter |
| GBR011 | 00037 | sativa | YUG | LUILBREG | | Winter |
| GBR011 | 01243 | sativa | GBR | CASTLETON POTATO | 1935 | Spring |
| GBR011 | 02214 | strigosa | GBR | TIREE OAT | 1949 | Spring |
| GBR016 | Lennon | sativa | GBR | Lennon | | Spring |

| | | | | | | |
|---|---|---|---|---|---|---|
| GBR016 | Millennium | sativa | GBR | Millennium | 2000 | Winter |
| HUN003 | RCAT010895 | sativa | HUN | Szegedi | 1964 | |
| HUN003 | RCAT012421 | sativa | HUN | Kulsovati B tf. | 1976 | |
| HUN003 | RCAT012599 | sativa | FIN | Sisu | 1948 | |
| HUN003 | RCAT012607 | sativa | HUN | V.Kalbens Vienauer | 1903 | |
| HUN003 | RCAT013381 | sativa | HUN | Monostorpalyi tf. | 1976 | |
| HUN003 | RCAT013388 | sativa | HUN | Anarcsi tf. | 1976 | |
| HUN003 | RCAT013398 | sativa | HUN | Pusztafoldvari tf. | 1976 | |
| IRL029 | 18 | sativa | IRL | GLASNEVIN TRIUMPH | 1885 | |
| IRL029 | 3 | sativa | IRL | Ayr Ally | 1959 | |
| IRL029 | 6 | sativa | IRL | Glasnevin Ardri | 1932 | |
| ITA037 | 10 | sativa | ITA | AVA | 1969 | |
| ITA037 | 47 | sativa | ITA | MONTAGNANA | 1979 | |
| ITA037 | 605 | sativa | ITA | DONATA | 1999 | |
| ITA037 | 683 | sativa | ITA | Primula | 2002 | Spring |
| ITA037 | 690 | sativa | ITA | Bionda | 2003 | |
| ITA037 | 695 | sativa | ITA | Teo BD40 | 2003 | |
| ITA037 | 700 | sativa | ITA | Genziana | 2004 | |
| ITA037 | 708 | sativa | ITA | Novella Antonia | 2005 | |
| ITA037 | 8 | sativa | ITA | ARGENTINA | 1969 | |
| LTU001 | 1114 | sativa | LTU | 1404-11 | 2000 | |
| LTU001 | 25 | sativa | LTU | Jaugila | 1994 | |
| LVA010 | LVA00027 | sativa | LVA | Stendes Velas | 1999 | |
| NLD037 | CGN03419 | sativa | NLD | Zwarte President | 1935 | |
| POL003 | PL50406 | sativa | POL | PULAWSKI SREDNIORYCHLY | 1919 | |
| POL003 | PL50528 | sativa | POL | GORSKI BIALY | 1976 | |
| POL003 | PL50731 | sativa | POL | PLATEK | 1974 | |
| POL003 | PL50951 | sativa | USA | | 1923 | |
| POL003 | PL50976 | sativa | POL | TEODOZJA | | |
| POL003 | PL51193 | abyssinica | | | 2000 | |
| POL003 | PL51229 | sativa | POL | PROPORCZYK | 1953 | |
| POL040 | Arab | sativa | POL | Arab | 2004 | |
| POL040 | Breton | sativa | POL | Breton | 2008 | |
| POL040 | Hetman | sativa | POL | Hetman | 2003 | |
| POL040 | Zuch | sativa | POL | Zuch | 2008 | |
| POL054 | Krezus | sativa | POL | Krezus | 2005 | Spring |

| | | | | | | |
|---|---|---|---|---|---|---|
| POL054 | Sam | sativa | POL | Sam | 1999 | |
| PRT005 | 2416 | sativa | PRT | | | |
| ROM007 | ROM007-15135 | sativa | ROM | Lapustesti | | |
| ROM007 | ROM007-16701 | sativa | ROM | Mures | | |
| ROM007 | ROM007-5087 | sativa | ROM | SACEL | | |
| ROM007 | ROM007-5938 | sativa | ROM | LUNCA ILVEI 2 | | |
| ROM007 | ROM007-9193 | sativa | ROM | BAISOARA 1 | | |
| RUS001 | 200105652 | sativa | RUS | | 1928 | |
| RUS001 | 200107022 | sativa | RUS | | 1928 | |
| RUS001 | 200107910 | sativa | RUS | | 1931 | |
| RUS001 | 200109633 | sativa | BYS | MULYAT | 1939 | |
| RUS001 | 200110507 | byzantina | ROM | AVENA AMARILLA | 1950 | Spring |
| RUS001 | 200110788 | sativa | MDA | Beltckii 1 | 1953 | Spring |
| RUS001 | 200110905 | sativa | RUS | Kinelskii | 1956 | Spring |
| RUS001 | 200111466 | sativa | RUS | | 1967 | |
| RUS001 | 200111655 | sativa | GRC | | 1970 | |
| RUS001 | 200113379 | sativa | ISL | | 1980 | |
| RUS001 | 200113917 | sativa | RUS | Signal | 1986 | |
| SVK001 | 1005 | sativa | SVK | Vendelin | | |
| SVK001 | 19903 | sativa | HUN | Gagybatory K Tajfajta | 2003 | |
| SVK001 | 20063 | sativa | SVK | Detvan | | |
| SVK001 | Zvolen | sativa | CZE | Zvolen | 2004 | |
| SWE002 | NGB2710 | sativa | SWE | Guldregn Ii | 1927 | |
| SWE002 | NGB5399 | strigosa | SWE | PURRHAVRE | 1991 | |
| SWE002 | NGB6185 | sativa | SWE | Fyris | 1911 | |
| SWE002 | NGB8759 | strigosa | DNK | Purhavre Fra Bur | 1991 | |
| SWE002 | NGB8760 | sativa | NOR | Nidar II | 1938 | |
| SWE002 | NGB9722 | sativa | DNK | Hedehavre Lyngby | 1913 | Spring |
| SWE006 | Cilla | sativa | SWE | Cilla | 1998 | |
| SWE006 | CPVO19960125 | sativa | SWE | BELINDA | 1997 | |
| SWE006 | SW Ingeborg | sativa | SWE | SW Ingeborg | 2007 | |
