# Supplementary figures and images for: Population structure and genome-wide association analysis for frost tolerance in oat using continuous SNP array signal intensity ratios
Source: Theor Appl Genet. 2016 Jun 18;129:1711–24. doi: 10.1007/s00122-016-2734-y (PMC4983288; doi:10.1007/s00122-016-2734-y)

**a**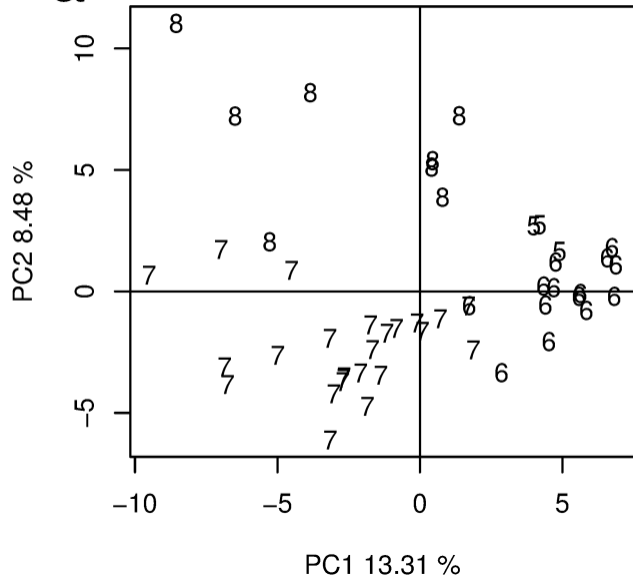**b**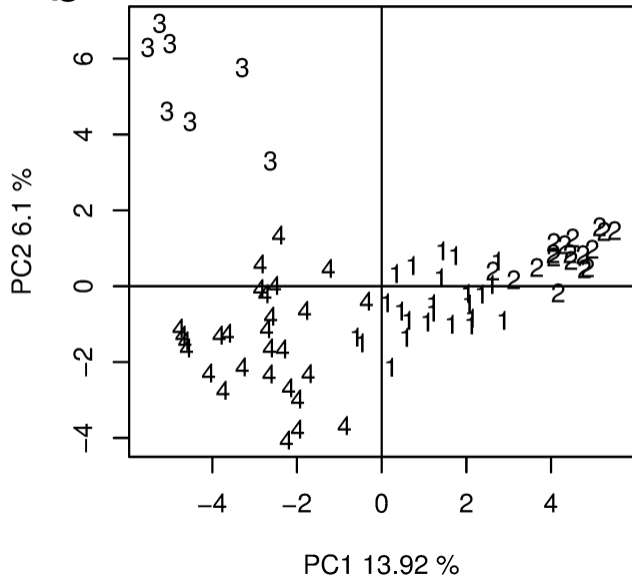

Supplement: Supplementary file 2 — OR02. PCA plots based on 3567 SNPs for the two main clusters detected by Ward’s method: (a) group A and (b) group B. Accessions are represented by numbers indicating Ward subgroup assignments (PDF 32 kb) [file 122_2016_2734_MOESM2_ESM.pdf]

# AVEQ08

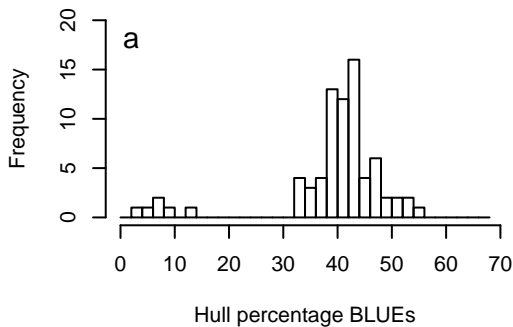

# AVEQ09

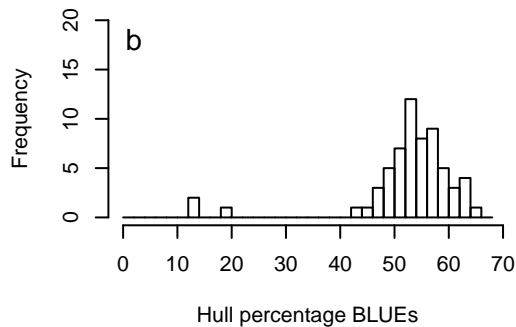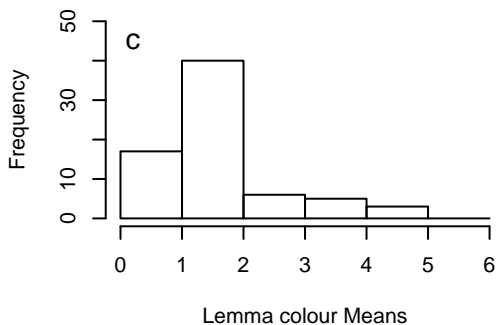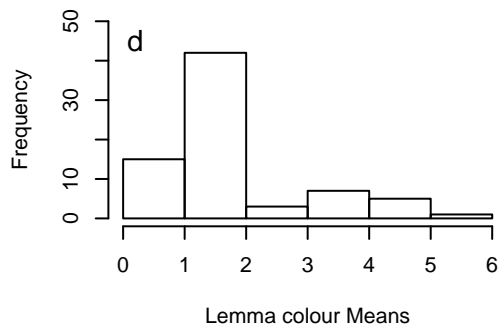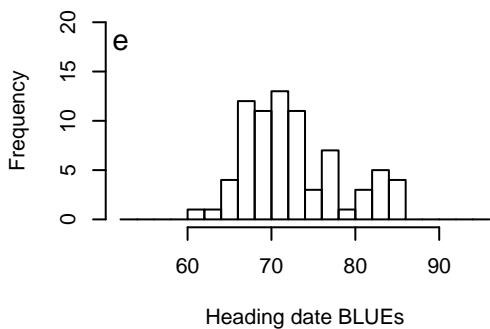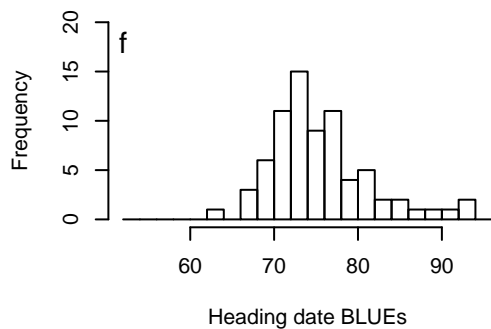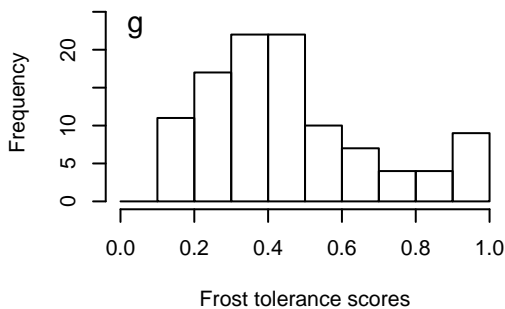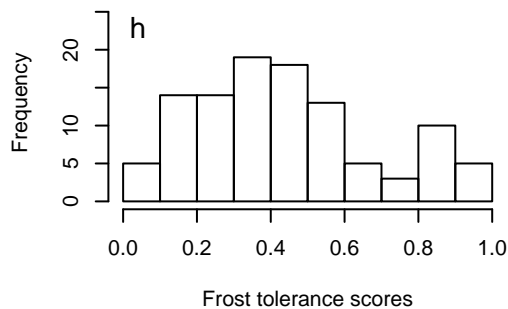

Supplement: Supplementary file 3 — OR03. Frequency distribution of the phenotypic variables used for GWAS. Lemma colour accession means (c), accession adjusted values for hull percentage (a) and heading date (e) and frost tolerance scores (g) in AVEQ08 and AVEQ09 (d, b, f, h, respectively) are shown (PDF 6 kb) [file 122_2016_2734_MOESM3_ESM.pdf]

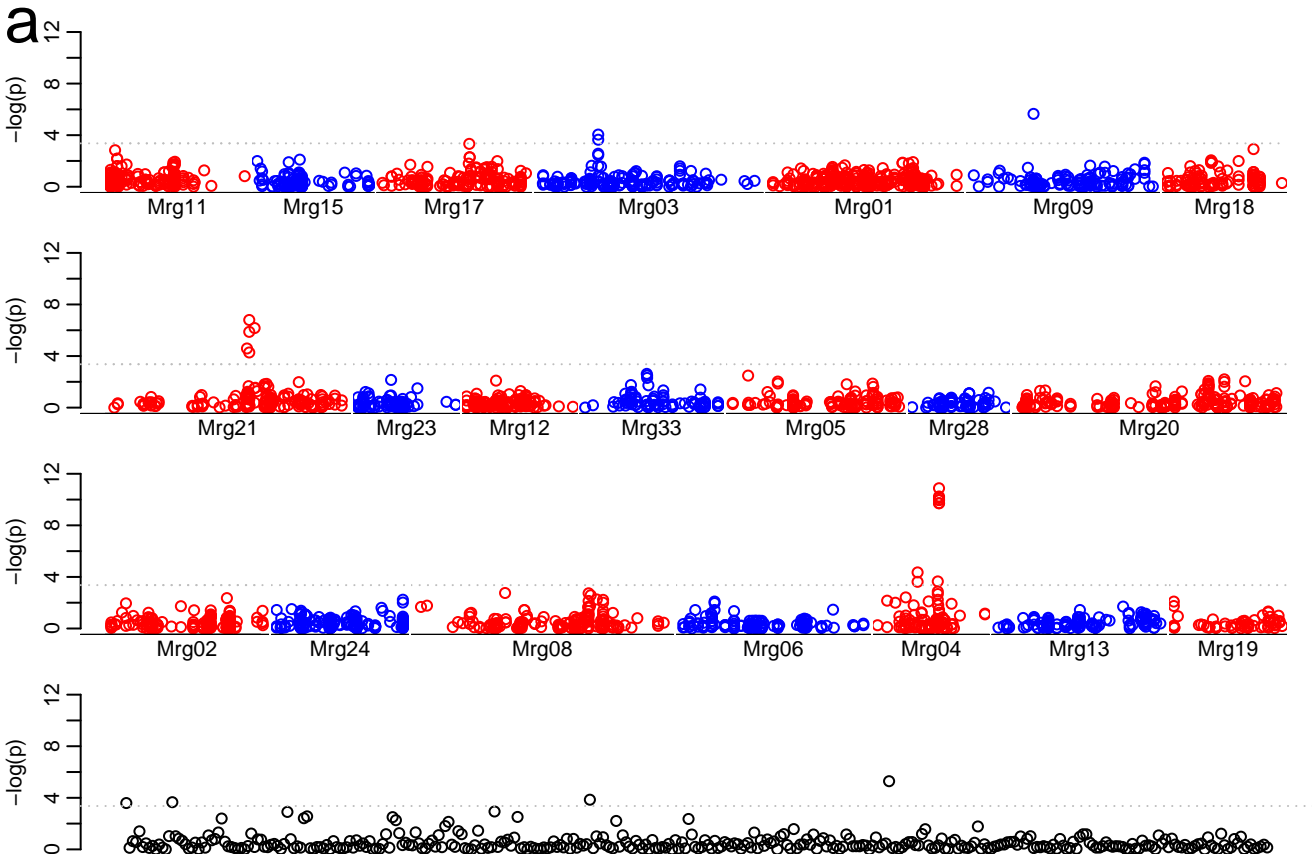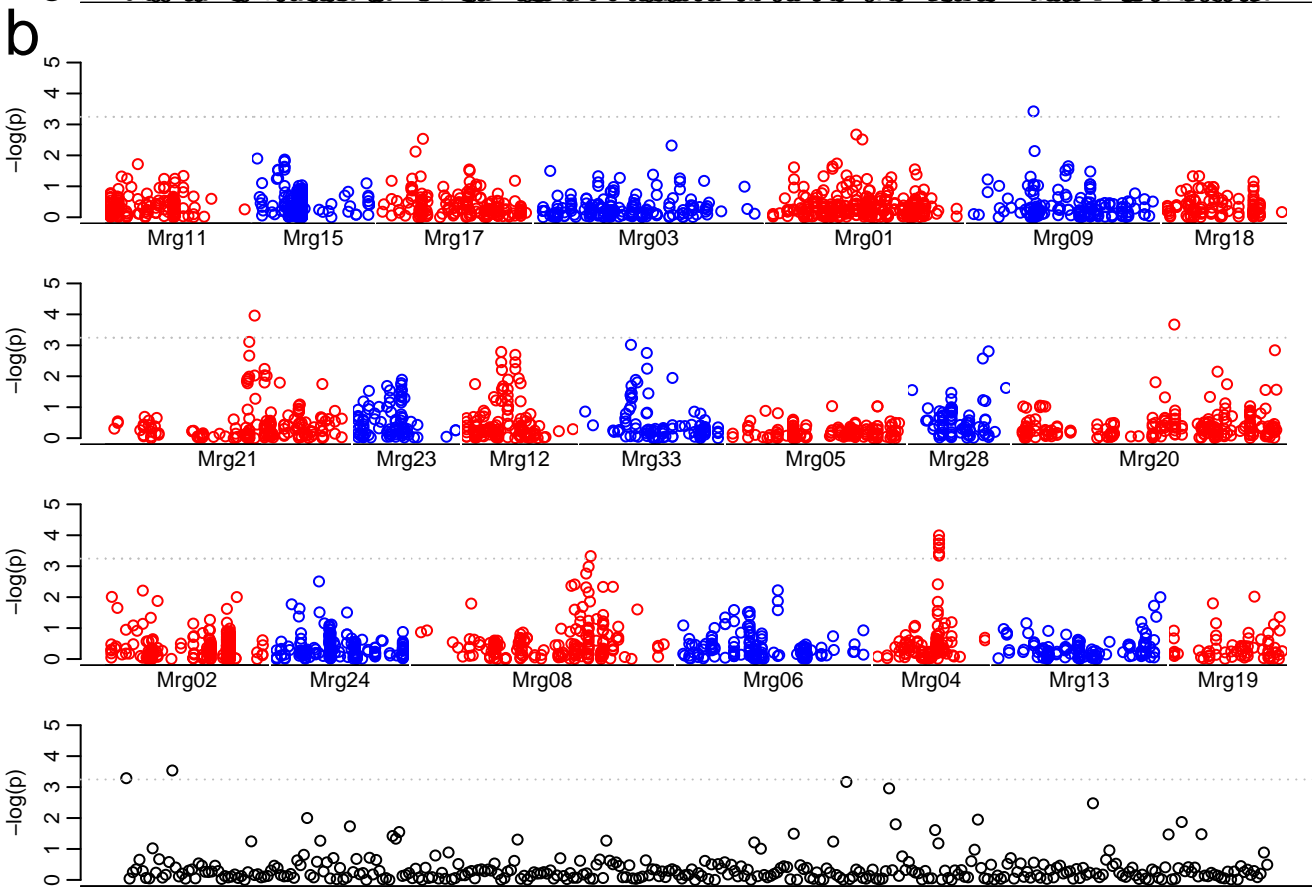

Supplement: Supplementary file 4 — OR04. Manhattan plots of –log P values (ordered by chromosome) calculated by GWAS for hull percentage in AVEQ08 (a) and AVEQ09 (b). Genetic position of markers is relative to the most recently available consensus map for oat (Chaffin et al. 2016). Black points indicate unmapped markers. The horizontal dotted line represents the genome-wide significance threshold (PDF 44 kb) [file 122_2016_2734_MOESM4_ESM.pdf]
