# Supplementary material for: Population structure and genome-wide association analysis for frost tolerance in oat using continuous SNP array signal intensity ratios
Source: Theor Appl Genet. 2016 Jun 18;129:1711–24. doi: 10.1007/s00122-016-2734-y (PMC4983288; doi:10.1007/s00122-016-2734-y)
Supplement: Supplementary file 5 — OR05. List of associated markers for lemma color in AVEQ08 and AVEQ09 (DOCX 15 kb) [file 122_2016_2734_MOESM5_ESM.docx]

| AVEQ08 | Locus_Name | -logP | Group | Position | Chrom |
| --- | --- | --- | --- | --- | --- |
|  | GMI_DS_LB_10098 | 4.26 | Mrg03 | 56.2 | 4C |
|  | GMI_ES05_lrc18884_211 | 4.31 | Mrg17 | 84.3 | 3C |
|  | GMI_ES15_c2369_181 | 6.20 | Mrg20 | 14.7 | 19A |
|  | GMI_ES_LB_8315 | 5.23 | NA | NA | NA |
|  |  |  |  |  |  |
|  |  |  |  |  |  |
| AVEQ09 | Locus_Name | -logP | Group | Position | Chrom |
|  | GMI_ES17_c5643_361 | 6.06 | Mrg02 | 27.4 | 9D |
|  | GMI_ES02_c19247_238 | 3.44 | Mrg03 | 3.5 | 4C |
|  | GMI_ES01_c13022_93 | 4.58 | Mrg03 | 5.1 | 4C |
|  | GMI_ES14_c986_797 | 3.40 | Mrg04 | 32.7 | 18D |
|  | GMI_ES01_c1307_465 | 3.82 | Mrg06 | 45.2 | 14D |
|  | GMI_ES14_c9429_338 | 5.78 | Mrg12 | 35.1 | 13A |
|  | GMI_ES_LB_3596 | 4.10 | Mrg12 | 35.3 | 13A |
|  | GMI_ES05_c9748_437 | 3.33 | Mrg12 | 35.3 | 13A |
|  | GMI_ES15_c11747_354 | 4.43 | Mrg12 | 35.4 | 13A |
|  | GMI_GBS_83715 | 3.40 | Mrg19 | 70.1 | 21D |
|  | GMI_ES15_c2369_181 | 7.64 | Mrg20 | 14.7 | 19A |
|  | GMI_ES_LB_8315 | 5.04 | NA | NA | NA |
