# Supplementary material for: Population structure and genome-wide association analysis for frost tolerance in oat using continuous SNP array signal intensity ratios
Source: Theor Appl Genet. 2016 Jun 18;129:1711–24. doi: 10.1007/s00122-016-2734-y (PMC4983288; doi:10.1007/s00122-016-2734-y)
Supplement: Supplementary file 6 — OR06. List of associated markers for heading date in AVEQ08 and AVEQ09 (DOCX 12 kb) [file 122_2016_2734_MOESM6_ESM.docx]

| AVEQ08 | Locus_Name | -logP | Group | Position | Chrom |
| --- | --- | --- | --- | --- | --- |
|  | GMI_ES05_c16902_400 | 3.40 | Mrg01 | 117.9 | 5C |
|  | GMI_ES03_c4395_765 | 3.42 | Mrg02 | 33 | 9D |
|  | GMI_ES05_lrc25030_330 | 3.93 | Mrg13 | 58.6 | 20D |
|  |  |  |  |  |  |
|  |  |  |  |  |  |
| AVEQ 09 | Locus_Name | -logP | Group | Position | Chrom |
|  | GMI_GBS_49707 | 3.94 | Mrg20 | 18 | 19A |
|  | GMI_ES_LB_9185 | 4.31 | Mrg20 | 115.6 | 19A |
|  | GMI_ES02_c16331_464 | 3.35 | Mrg21 | 122.8 | 8A |
