# Supplementary material for: Population structure and genome-wide association analysis for frost tolerance in oat using continuous SNP array signal intensity ratios
Source: Theor Appl Genet. 2016 Jun 18;129:1711–24. doi: 10.1007/s00122-016-2734-y (PMC4983288; doi:10.1007/s00122-016-2734-y)
Supplement: Supplementary file 7 — OR07. GWAS observed p -values versus expected p -values (Quantile–quantile plot) for frost tolerance (a for AVEQ08 and b for AVEQ09), heading date (c for AVEQ08 and d for AVEQ09), lemma colour (e for AVEQ08 and f for AVEQ09), and hull percentage (g for AVEQ08 and h for AVEQ09). The red line represents the p -values distribution for simple association and the blue line represents the p -values for the model that corrects for kinship using a subset of 302 uniformly spaced markers (PDF 365 kb) [file 122_2016_2734_MOESM7_ESM.pdf]

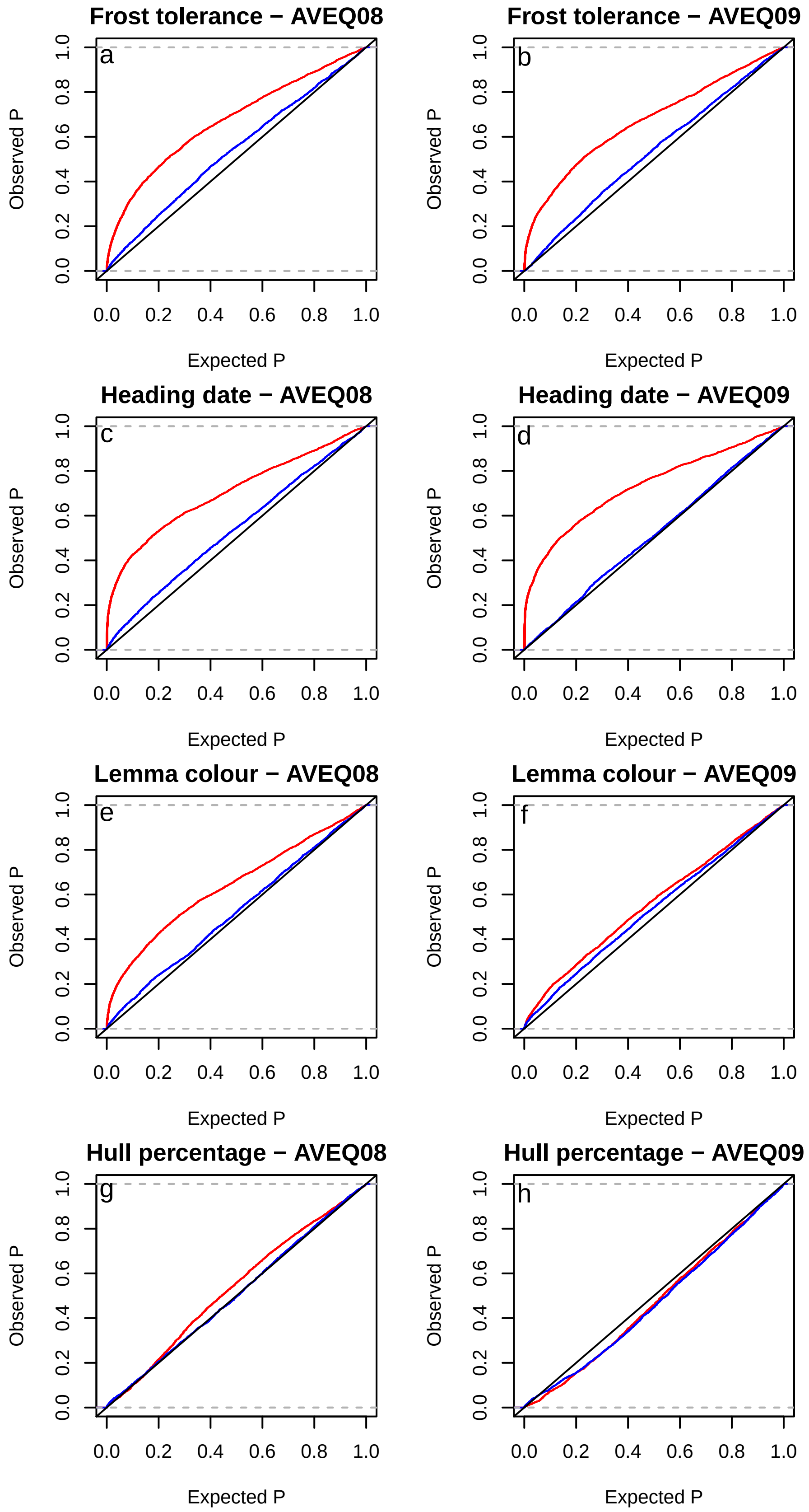
Frost tolerance − AVEQ08
Frost tolerance − AVEQ09
Heading date − AVEQ08
Heading date − AVEQ09
Lemma colour − AVEQ08
Lemma colour − AVEQ09
Hull percentage − AVEQ08
Hull percentage − AVEQ09
a
b
c
d
e
f
g
h
Observed P
Expected P
0.0
0.2
0.4
0.6
0.8
1.0
